# Robust fetoscopic mosaicking from deep learned flow fields

**DOI:** 10.1007/s11548-022-02623-1

**Published:** 2022-05-03

**Authors:** Oluwatosin Alabi, Sophia Bano, Francisco Vasconcelos, Anna L. David, Jan Deprest, Danail Stoyanov

**Affiliations:** 1 University of Bordeaux, Bordeaux, France; 2 Wellcome/EPSRC Centre for Interventional and Surgical Sciences (WEISS) and Department of Computer Science, University College London, London, UK; 3 Elizabeth Garrett Anderson Institute for Women’s Health, University College London, London, UK; 4 NIHR University College London Hospitals Biomedical Research Centre, London, UK; 5 Department of Development and Regeneration, University Hospital KU Leuven, Leuven, Belgium

**Keywords:** Optical flow, Fetoscopy, Video mosaicking, Twin-to-twin transfusion syndrome

## Abstract

**Purpose:**

Fetoscopic laser photocoagulation is a minimally invasive procedure to treat twin-to-twin transfusion syndrome during pregnancy by stopping irregular blood flow in the placenta. Building an image mosaic of the placenta and its network of vessels could assist surgeons to navigate in the challenging fetoscopic environment during the procedure.

**Methodology:**

We propose a fetoscopic mosaicking approach by combining deep learning-based optical flow with robust estimation for filtering inconsistent motions that occurs due to floating particles and specularities. While the current state of the art for fetoscopic mosaicking relies on clearly visible vessels for registration, our approach overcomes this limitation by considering the motion of all consistent pixels within consecutive frames. We also overcome the challenges in applying off-the-shelf optical flow to fetoscopic mosaicking through the use of robust estimation and local refinement.

**Results:**

We compare our proposed method against the state-of-the-art vessel-based and optical flow-based image registration methods, and robust estimation alternatives. We also compare our proposed pipeline using different optical flow and robust estimation alternatives.

**Conclusions:**

Through analysis of our results, we show that our method outperforms both the vessel-based state of the art and LK, noticeably when vessels are either poorly visible or too thin to be reliably identified. Our approach is thus able to build consistent placental vessel mosaics in challenging cases where currently available alternatives fail.

**Supplementary Information:**

The online version contains supplementary material available at 10.1007/s11548-022-02623-1.

## Introduction

Twin-to-twin transfusion syndrome (TTTS) is a severe complication of monochorionic twin pregnancies where both fetuses share the same placenta [6]. This condition occurs when abnormal placental vascular anastomoses on the chorionic plate of the placenta allow for the transfusion of blood disproportionately from one fetus to another [6]. TTTS occurs in about 1 in 2000 pregnancies and it can be life-threatening for both fetuses. The standard method for treating TTTS is a laser ablation image-guided procedure, termed as fetoscopic laser photocoagulation (FLP), that photocoagulates abnormal vascular anastomoses responsible for the complication. The process involves surgeons searching for abnormal vascular anastomoses using the fetoscope. The field of view of a fetoscope is limited compared to the area being operated, and this may lead to anastomoses being missed by the surgeon and incomplete treatment [19]. Other common problems encountered include unusual placenta position (anterior or posterior placenta), poor visibility, and limited maneuverability. Expanding the surgical field of view through automatic video frame registration and mosaicking can provide better visualization of the in utero scene and could support the surgeon in the identification of abnormal anastomoses during the laser procedure.

Various image mosaicking methods have been explored to provide an expanded view of the placenta from fetoscopic video frames and overcome the associated visibility and navigation challenges. Approaches mostly differ in how the alignment of consecutive frames is performed. Daga et al. [9, 26] utilized a classical pipeline for alignment of sparse point landmarks, relying on detection and matching of handcrafted features (SIFT). However, this approach only works reliably on synthetic phantom data and achieves a drastically lower performance with in vivo TTTS fetoscopic video due to multiple factors such as lower resolution, poor illumination, lack of texture, low contrast, specular reflections, occlusion by particles in the amniotic fluid [24].

On the other hand, dense registration methods have shown a significantly larger success in dealing with in vivo TTTS fetoscopic data than sparse feature-based methods. Peter et al. [24] proposed a direct pixel-wise alignment of gradient orientations and an optimization framework for ensuring long range consistency using bag-of-words. However, the method was designed and validated only on a single in vivo video clip. Tella-Amo et al. [30] used an electromagnetic tracker with the fetoscope on an ex-vivo setup. They proposed a dense registration mosaicking method capable of correcting for drift, but this hardware setup has not yet been replicated in a real surgery scenario, which can be challenging both in terms of reliable calibration and regulatory approval.

Deep learning techniques have also been used to estimate motion model parameters in fetoscopy with success. DeTone et al. [10] presented a deep learning regression approach to calculate the geometric transformation between two images. This method does not require local feature detection or pixel-based alignment algorithms. Bano et al. [3, 5] extended this approach to handle sequential data in fetoscopic videos by proposing controlled data augmentation and outlier rejection methods. The method [3, 5] outperforms feature-based mosaicking and shows reliable results on a larger set of placental videos. However, it suffers from drifting error in the case of non-planar views and occlusions. A recent approach based on the registration of vessel segmentation by Bano et al. [4] has shown for the first time reliable mosaics on multiple in vivo sequences with significantly different visual appearances. This approach, however, is expected to fail when vessel segmentation is inaccurate or its shape is not discriminative enough for sequential registration. Potential causes for segmentation failure include scarce or thin vasculature and specular reflections, which we experimentally confirm in “Experimental Analysis”.

Optical flow is a well-established technique in computer vision that attempts to match all corresponding pixels between two consecutive frames based on local similarity [13]. This is a viable alternative for fetoscopic image registration that does not explicitly rely on vessel features and therefore can overcome the above-mentioned challenge. However, classic optical flow approaches such as pyramidal Lucas–Kanade (LK) are not reliable enough on fetoscopic data as demonstrated in [4]. Better results have been obtained by modifying the cost function of LK to be more sensitive to vessel structures [24], but this undermines our goal of dealing with images where such vessels are not clearly visible. More recent state-of-the-art optical flow methods such as DeepFlow [32], FlowNet [11], FlowNet-2 [15], PWC-Net [28], and RAFT [29] are based on deep learning networks and to the best of our knowledge they have not been tested before as a backbone for fetoscopic mosaicking. While these methods have shown impressive results in other computer vision domains, there are particular challenges in their application to fetoscopic data, with the most predominant being the presence of floating particles and specular reflections that are inconsistent with global camera motion. One potential solution would be to fine-tune the optical flow network parameters on fetoscopic data, however, this is not possible to achieve at the moment since there is no available camera motion groundtruth for in vivo fetoscopic data.

**Fig. 1:**
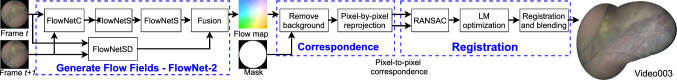
An overview of the proposed framework which is composed of a flow field generation block that provides features, a pixel correspondence block that performs feature matching, and a registration block that generates mosaic through LM optimization

This paper proposes a fetoscopic video mosaicking approach by combining deep learning-based optical flow with robust RANSAC [12] estimation for filtering inconsistent motions, thus providing a reliable pixel-alignment solution that is able to deal with floating particles and reflections which are not consistent with fetoscopic camera motion. The proposed fetoscopic mosaicking pipeline uses state-of-the-art optical flow as a backbone and reliably works regardless of whether vessels are clearly visible within the fetoscopic field of view or not. We can summarize our contributions as follows:

1. We propose a new fetoscopic mosaicking pipeline that relies on optical flow (FlowNet-2 [15]), robust estimation (RANSAC), and local refinement (Levenberg–Marquardt [23]) for incremental camera motion estimation. Unlike the current state of the art [4], the approach does not explicitly rely on clearly visible vessels.
2. We experimentally validated our approach on 6 in vivo TTTS video sequences, which are an extended version of the publicly available fetoscopy placenta dataset^1^. The extended dataset used in our experimentation has been made available, under the fetoscopy placenta dataset webpage, for reproducibility.
3. We show that FlowNet-2 pretrained on non-medical data reliably initializes fetoscopic mosaics, provided that inconsistent motions due to floating particles and specularities are identified and filtered with RANSAC. Note that FlowNet-2 cannot be fine-tuned in a supervised way on fetoscopic data due to the lack of groundtruth. While self-supervised fine-tuning [18] could be an option, our experimental results show that pretrained models are sufficiently reliable for fetoscopic mosaicking.
4. We experimentally justify the components of our pipeline through direct experimental comparison against relevant alternatives. The choice of FlowNet-2 is compared against the recently proposed Recurrent All-Pairs Field Transforms for Optical Flow (RAFT) [29] and the classic pyramidal Lucas–Kanade (LK) [20]. The choice of RANSAC for robust optimization is compared against direct iterative optimization using a robust metric [31].
5. We show that our approach reliably tracks the camera motion in cases where the vessel-based state of the art for fetoscopic mosaicking [4] fails due to unreliable vessel features, while keeping a similar or better performance when vessels are clearly visible. As a consequence, we are able to track camera motion and produce consistent fetoscopic mosaics in more scenarios and with longer video sequences.

## Our method

Our proposed mosaicking framework estimates the optical flow between consecutive frames using FlowNet-2 [15] to establish pairwise point correspondences for all available pixels. Outlier pixels inconsistent with a global affine transformation are detected with Random Sample Consensus (RANSAC), and the re-projection error of inlier pixels is then minimized with iterative Levenberg–Marquardt (LM) optimization to obtain pairwise affine transformation estimations. Finally, a mosaic is incrementally built by left-hand matrix multiplication of pairwise affine transformations for obtaining the relative affine transformation for the entire video. The outline of our method is represented in Fig. 1.

### Point correspondences from optical flow

Optical flow aims at generating a 2D displacement vector field between a source and target images (i.e., a flow field). To perform optical flow between consecutive frames, we use the FlowNet-2 [15] a deep learning architecture, with its parameters pretrained on the flying chairs synthetic dataset [1]. While the training data is very far from representative of fetoscopic video appearance and characteristic motions, its very large size and accurate groundtruth enable training a network that focuses on capturing the fundamental geometric relationships between local appearance changes rather than learning application-specific priors. This has shown a great generalizability power in computer vision problems, and we show (in Sec. 3.4) that it is also a reliable backbone for fetoscopic video. While there are other potentially viable optical flow options, we have chosen FlowNet-2 since our experiments demonstrate it to be more reliable than alternative deep learning approaches such as RAFT [29] or classic approaches such as LK [20].

Once a flow field between consecutive frames is obtained, pairwise correspondences between every pixel in the first frame and image coordinates in the second frame can be established by adding the flow field displacement to the coordinate of every pixel in the first frame. Pixels outside the visible circular area of the fetoscopic image are masked out (as shown in Fig. 1). Given the position of each pixel $(x_i , y_i)$ in the $i^{th}$ frame and the flow vector $(u_i, v_i)$ for each pixel, the estimated pixel position $(x_{i+1}^{\prime }, y_{i+1}^{\prime } )$ in the next frame is given by:

1 $$\begin{aligned} (x_{i+1} ^{\prime }, y_{i+1}^{\prime }) = \alpha * [(x_i , y_i) + (u_i, v_i) ], \end{aligned}$$

where $\alpha \in {0,1}$ is a coefficient which is 1 when a pixel is in the circular Boolean mask of the fetoscope and 0 when it is outside the mask.

### Sequential registration

Similarly to previous works [4, 24], we formulate fetoscopic image registration as finding an affine transformation, ${\textbf{A}}$, between consecutive frames [4, 24]. We choose affine transformation instead of projective transformation because estimations are more stable and less prone to divergent shrinking or enlargement of the mosaic when accumulating relative transformations from a large number of consecutive frames. This is in line with the findings of [4, 24].

An affine transformation, ${\textbf{A}}$, between two consecutive frames is given by:

$$\begin{aligned} \begin{bmatrix} x_{i+1}' \\ y_{i+1}' \\ \end{bmatrix} \Longleftarrow {\textbf{A}} \begin{bmatrix} x_i \\ y_i \\ 1 \\ \end{bmatrix} \qquad {\textbf{A}} = \begin{bmatrix} a_{11} &{}&{} a_{12} &{}&{} b_1\\ a_{21} &{}&{} a_{22} &{}&{} b_2\\ \end{bmatrix} \end{aligned}$$

where $a_{11}, a_{12}, a_{21}, a_{22}$ are composed of scale, shear, and rotation transformation components in an affine transformation, and $b_1, b_2$ specifies the translation components.

Unlike these previous approaches, however, we establish explicit pairwise point correspondences $\{(x_{i}, y_{i}),(x_{i} ^{\prime }, y_{i}^{\prime } )\}$ between consecutive frames, and therefore we benefit from the extensive literature on estimating 2-view transformations from point matches [14, 25]. In our method, this means that a linear solution for an affine transformation can be found from 3 or more point correspondences. Given that outliers are present, we follow the well-established “gold standard” pipeline in multiple view geometry where the minimal least-squares solver [7] (3 points in our case) is used as a candidate solution generator within the RANSAC framework, followed by Levenberg–Marquardt algorithm for minimizing the squared sum of inlier point re-projection error:

2 $$\begin{aligned} E= & {} \sum _i( x_{i} - a_{11}x_{i}^{\prime } - a_{12}y_i^{\prime } - b_{1})^{2} \nonumber \\{} & {} + (y_{i} - a_{21}x_i^{\prime } - a_{22}y_i^{\prime } - b_2)^{2}. \end{aligned}$$

While this approach has been developed mainly with sparse point correspondences in mind, it has been shown to be equally applicable to dense pixel-wise correspondences [27]. An alternative approach to bypass RANSAC would be to use an iterative optimizer with a robust cost function instead of Eq. 2. However, this would mean that initialization would rely on a non-robust linear estimation that is more likely to be unreliable and lead to local minima.

Sequential registration for mosaicking is performed by selecting a frame (usually in the center of the mosaic) as the reference frame, which is also referred as the mosaic plane. All other frames are then warped forward or backward onto the reference frame by computing relative affine transformations through left-hand matrix multiplication of pairwise affine transformations. For generating a seam-free mosaic, the warped frames are blended using Enblend,^2^ which uses the Burt–Adelson multi-resolution spline algorithm.

## Experimental analysis

### Dataset description

**Table 1:** Summary of the existing fetoscopy placenta dataset [4] and the extended version of this dataset with longer duration of videos used in our paper

Seq.	Fetoscopy dataset (frames)	Extended dataset (frames)
Video 1	400	420
Video 2	200	300
Video 3	50	150
Video 4	100	200
Video 5	100	200
Video 6	100	200

For qualitative and quantitative analysis, we use the same fetoscopic dataset presented in [3] but with additional frames as shown in Table 1. The extended dataset will be released with this publication. The dataset consists of 6 in vivo fetoscopic video sequences taken from different TTTS laser therapy surgeries. Each video sequence has varying conditions of occlusion, texture, lighting, and floating particles. The first column in Table 1 shows the number of frames in the original dataset from [3], while the last column shows the number of frames in the extended dataset.

### Evaluation metric

Since the groundtruth transformations are not available, we use the quantitative metric described in [2, 4] for quantifying accumulated drift error within *N* frame intervals. This is done by computing the structural similarity index metric (SSIM) between a frame *i* and a warped source frame $i+t$, with $t \in \{1,2,\ldots ,N\}$. Since the warping is built incrementally from consecutive frame transformations, drift error is accumulated with increasing *t*. Similar to [2, 4] we use $N=5$. Similar to [2, 4] We visualize the SSIM calculated as a standard boxplots which contains the median, 1st Quartile(Q1),3rd Quartile(Q3), a Minimum Value $(Q1- 1.5*IQR)$, a maximum value $(Q3+ 1.5*IQR)$, and outliers which lie above the Maximum value and below the minimum value. Using the boxplots of the SSIM, we can evaluate the performance of various methods by comparing their distribution. We are especially concerned with the “outliers on the minimum side” as it indicates failures in registration.

**Fig. 2:**
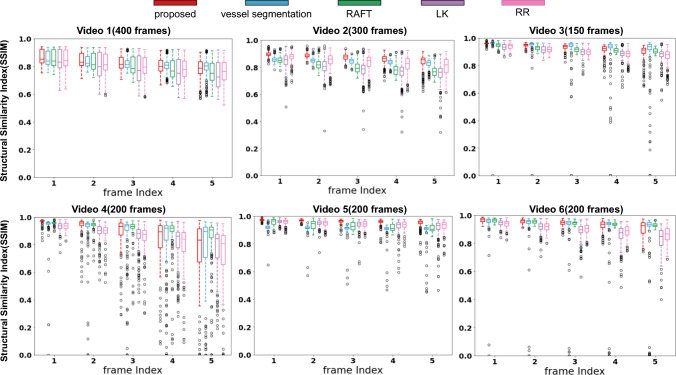
Quantitative comparison of the proposed (red), vessel segmentation-based (blue), RAFT backbone (green) and LK-based (purple), RR (light purple) methods using the drift analysis metric from [4]

### Comparison methods

We compare our method against the complete pipeline of the current state-of-the-art vessel-based approach presented in [4], and classic Lucas–Kanade (LK) pyramidal registration. We perform an ablation study of different components of our own pipeline. We replace FlowNet-2 with Recurrent All-Pairs Field Transforms (RAFT) [29] as the Optical Flow backbone. Note that RAFT has been recently reported as a top performer on widely popular optical flow benchmarks such as Sintel [8] and KITTI [22]. We replace LM+RANSAC with robust nonlinear regression method for utilizing a smooth approximation function of the absolute loss(soft l1 loss) [31] as our robust registration (RR) method.

Additionally, we investigate the outliers detected by RANSAC to show their correlation with the frequent floating particles visible in fetoscopic videos and identify situations where vessel segmentation clearly fails, showcasing where our method demonstrates clear advantage.

All experiments were done in python using NVIDIA k80 GPUs from Google Colaboratory. FlowNet-2 [15] was used with pretrained weights provided by the authors.^3^ The datasets used to obtain the pretrained weights were flyingChairs [11], flyingThings [21], and ChairsSDHoM [15]. RAFT [29] was used with pretrained weights provided by the authors.^4^ The datasets used obtain the pretrained weights were flyingChairs [11], flyingThings [21], Sintel [8], KITTI [22] and HD1K [16] datasets. RANSAC threshold used for all sequences was

3 $$\begin{aligned} \left|\left|P_D - P_R \right|\right|_2 \le 6 \end{aligned}$$

where $P_D$ are the destination points and $P_R$ are the re-projected points. This was determined empirically by viewing removed pixels.

**Fig. 3:**
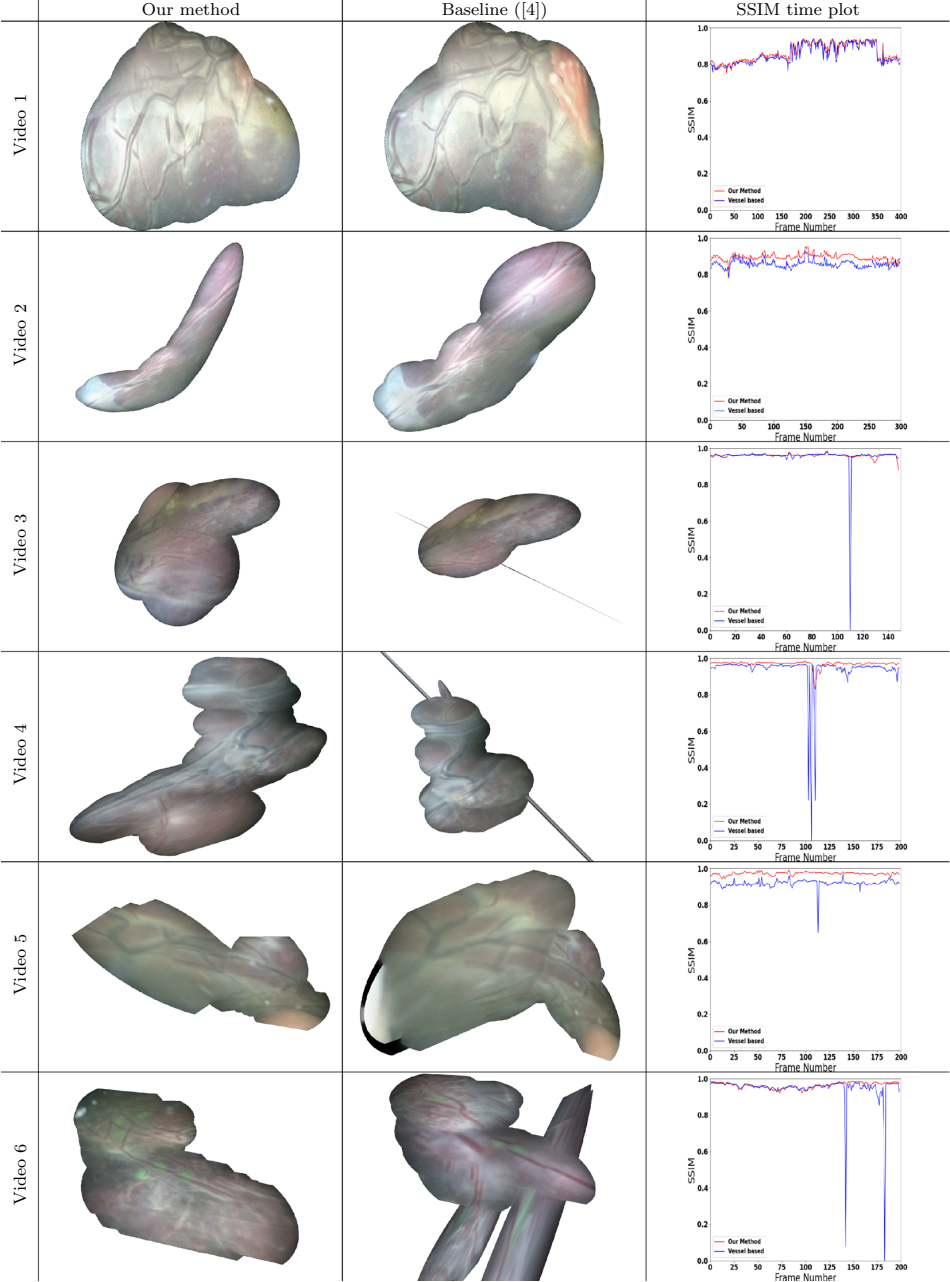
Visualization of the mosaics produced by our proposed method. The first column shows final mosaics on various sequences from the extended dataset using our method while the second column show final mosaics on the same sequences using the state-of-the-art approach from [4]. The third column shows SSIM time plots which plot the SSIM of the registration of consecutive images. The red colored plot is our method while the blue is the baseline (Table 2). Notice that the tracking fails in Videos 3, 4, 6 in the case of [4] after frame number 113, 106, 140. While our proposed method resulted in consistent mosaics for the complete duration of all extended videos

**Table 2:** Mean of the structural similarity index metric (SSIM) between frame *i* and the warped source frame $i + t$, with $t \in \{1, 2,\ldots , N\}$. The bold values represent the method with a higher SSIM score

	Video 1		Video 2		Video 3		Video 4		Video 5		Video 6	
	Ours	[4]	Ours	[4]	Ours	[4]	Ours	[4]	Ours	[4]	Ours	[4]
Mean t = 1	**0.8659**	0.8543	**0.8954**	0.8569	**0.9594**	0.9552	**0.9699**	0.9386	**0.9719**	0.9182	**0.9642**	0.9465
Mean t = 2	**0.8435**	0.8355	**0.8841**	0.8494	**0.9473**	0.9389	**0.9375**	0.9094	**0.9676**	0.912	**0.9563**	0.9295
Mean t = 3	**0.8232**	0.8217	**0.8722**	0.8431	**0.9318**	0.9152	**0.8866**	0.8665	**0.9629**	0.9078	**0.9456**	0.9129
Mean t = 4	0.8045	**0.8094**	**0.8566**	0.8358	**0.912**	0.8888	**0.8269**	0.8167	**0.9563**	0.9021	**0.9276**	0.8959
Mean t = 5	0.7868	**0.7978**	**0.8387**	0.8267	**0.8894**	0.8615	**0.7657**	0.7606	**0.948**	0.8955	**0.9042**	0.8795

**Fig. 4:**
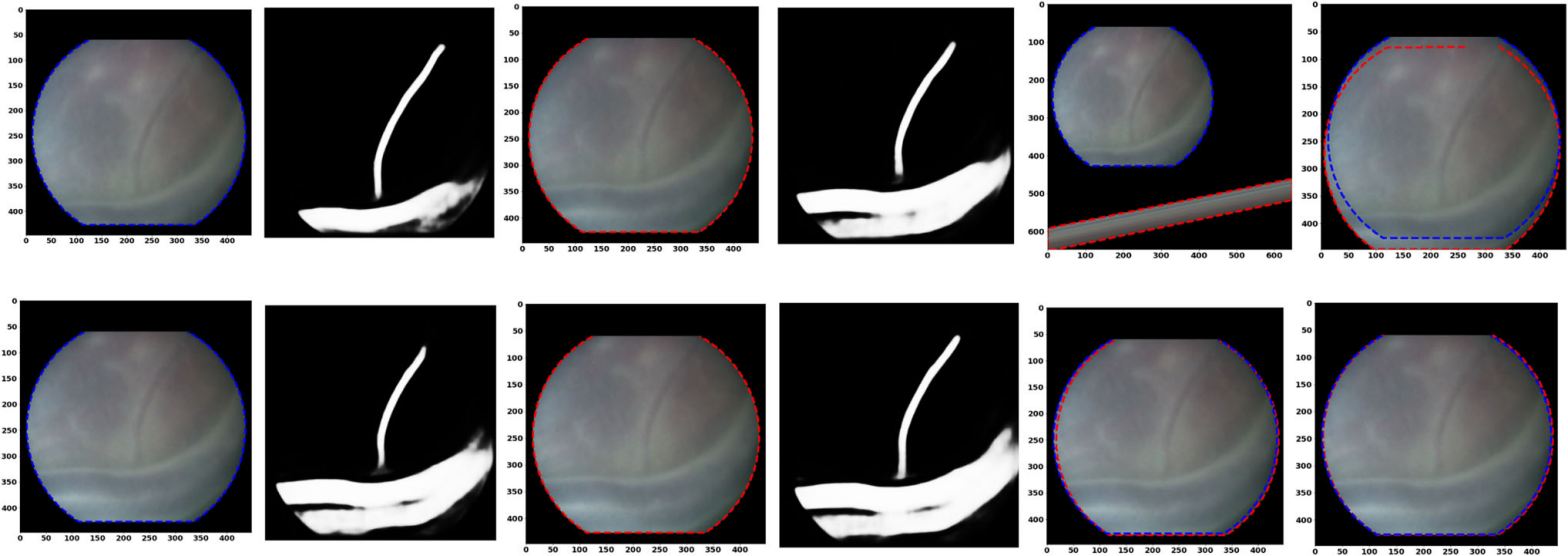
Vessel-based method qualitative analysis using Video 4. Top row shows when vessel segmentation method fails in registration (at frame 106). Bottom row shows vessel segmentation with good registration(at frame 99 highest SSIM). From left to right : destination image, destination image vessels segmentation, source image, source image vessels segmentation, registration using vessel segmentation, registration using our method

### Results and discussion

We perform both quantitative and qualitative comparison of our proposed method with the existing alternatives. Quantitative drift error results are presented in Fig. 2 which displays the error bar for up to 5 frame SSIM metric (as discussed in Sec. 3.2) for the proposed method (red), its RAFT variant (green), its robust regression variant - RR (light purple), LK-based (purple) and current state-of-the-art vessel segmentation-based (blue) methods. In Video 1, since vessels are clearly visible throughout the entire sequence, and therefore all methods perform relatively well, with a slight disadvantage to the classic LK approach. In the remaining videos, our approach (red) is consistently better than the vessel-based state of the art. We highlight that in Fig. 2 the individually plotted dots (outliers) on the lower bottom of the plots indicate cases of clear failure in registration. It is noteworthy that our proposed method, its RAFT variant, and the RR variant are the only ones where these clear failures do not occur in any of the sequences. We also note that to perform evaluation using RAFT and RR, we use the same pipeline described in Section 2, but we replace FlowNet-2 with RAFT and LK+RANSAC with a RR method. Such failures, even if they happen on a single frame window, can invalidate the addition of all subsequent frames to a mosaic due to loss of camera motion tracking. LK performance is comparatively poor in most of the videos with lower similarity scores, larger variation, and more failure cases. The median similarity score of the vessel-based method is overall just slightly lower or comparable with our proposed method, but very low similarities (outliers) in just a few frames (seen in Videos 3, 4, 5, 6) result in severely distorted or inconsistent mosaic reconstructions, as evident from Fig. 3.

In addition, we compare the results of the baseline method (vessel based) and our method. We computed the mean of the structural similarity index metric (SSIM) between a frame *i* and a warped source frame $i+t$, with $t \in \{1,2,\ldots ,N\}$, as a measure of central tendency. We clearly see that the values for our method are better than the baseline.

**Fig. 5:**
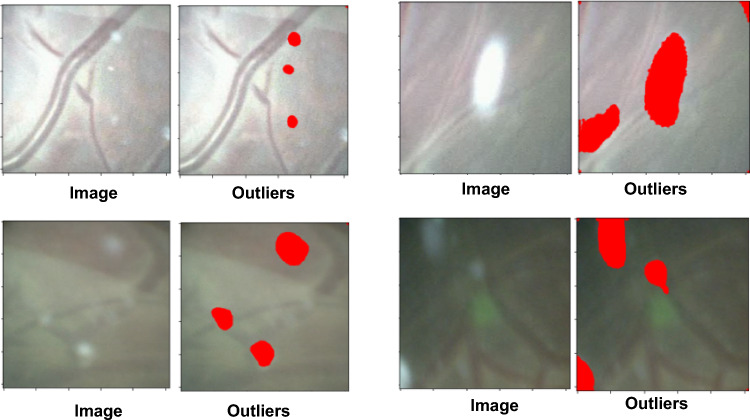
Examples of outlier regions (in red) detected by RANSAC. Outliers generally correspond to floating particles and bright specular reflections(white and bright spots on the Image) inconsistent with fetoscope motion. Images obtained from Video 1 (top-left), Video 2 (top-right), Video 5 (bottom-left), Video 6 (bottom-right)

The qualitative results^5^ visually comparing the mosaics generated from the proposed and vessel-based methods are presented in Fig. 3. Video 1 and Video 2 are examples where no clear failures happen and both our method and the vessel-based approach produce reasonably consistent mosaics. On the other hand, Video 3 and Video 4 represent cases where our method performs better, but the vessel-based approach stopped working after the first clear registration failure happens at frame number 113 and 106, respectively. Video 4 is the most challenging sequence for all methods and even if our approach is able to produce a coherent mosaic for the entire sequence, it still has some noticeable drift after a considerable amount of frames. In Video 5, our proposed method also works better as it has less drift compared to the vessel-based method. The drift in the vessel-based method is noticeable in Video 5 from frame 116, as there is a rapid shrinking between consecutive frames and again at frame 160 where there is a rapid enlargement in consecutive frames. This is because of the failures in registration in vessel-based method. Our method did not experience rapid shrinking or enlargements at these frames. In Video 6, the vessel segmentation method looses tracking after frame 145, while our proposed method does not lose tracking for the entire duration of the sequence.

Investigating further the failure cases in vessel-based method, we visualize in the top row of Fig. 4 registration failure for video 4 which is at frame 106. This can be seen from the SSIM time plots in Fig. 3. In addition, we visualize a successful registration (highest SSIM) at frame 99 from Video 4 on the bottom row of Fig. 4. This figure shows that vessel segmentation is unreliable in cases where vessels are extremely thin, sparse, or blurry. It can be noticed in the top row of Fig.  4 that there is a vessel which is not detected completely in the destination image, but detected correctly in the source image. The registration using vessel segmentation for this pair of source and destination images is 0.00048, approximately 0, as the registration failed for this pair. While in the bottom row of Fig.  4, registration 99 is at a similar position as registration 106, and we notice that the vessel was well detected in frame 99, hence the SSIM was 0.967643 which is very good. During the failure case in the top row of Fig. 4, we show that our method accurately registers this frame, and our method as works well in the bottom row.

Finally, we take a closer look at the effects of RANSAC on our pipeline in Fig. 5, where we plot the flow vectors which are filtered out as outliers. We observe that this generally corresponds to motions that are in a different direction to the global camera motion, as in the case of floating particles and bright specular reflections. Utilizing pixels from these floating particles and specular reflections would lead to poorer affine transformation estimation. This further validates the results obtained by our proposed method and its robustness against outliers, which contributes toward minimizing failures.

## Conclusion

We propose a framework for generating mosaics from fetoscopic videos. We generate dense flow fields produced using pretrained FlowNet-2. The dense flow fields are used to establish pairwise point correspondence. This is combined with robust outlier filtering with RANSAC and iterative refinement with the Levenberg–Marquardt. Our final mosaic is built using all the pairwise affine transformations obtained. To the best of our knowledge, this is the first solution that does not rely on explicit vessel alignment to demonstrate consistent mosaics in several in vivo fetoscopic sequences with varying appearance. While previous attempts at utilizing a non-vessel approach either fail (sparse feature matching) or perform poorly (standard optical flow), we show that combining modern deep learning optical flow with classic robust estimation produces reliable fetoscopic mosaics. When compared against the vessel-based state of the art, our approach demonstrates its main advantage in reliably dealing with videos that contain sequences where vessels are either sparse or not clearly visible and thus vessel detection fails. Our method thus is able to build consistent seam-free mosaics in a larger set of scenarios, and from larger uninterrupted sequences of fetoscopic video. As future work, we plan to design a hybrid solution that switches between a vessel-based approach and optical flow depending on the most appropriate context and to develop reliable selection mechanisms. In addition, we plan to utilize methods for global optimization and reduction of error drift such as [17], to provide long-term consistency in large-scale fetoscopic mosaicking.

### Supplementary Information

Below is the link to the electronic supplementary material.

Supplementary file 1 (mp4 29186 KB)

## Footnotes

1 Fetoscopy placenta dataset from [4]: https://www.ucl.ac.uk/interventional-surgical-sciences/fetoscopy-placenta-data.

2 Enblend: http://enblend.sourceforge.net.

3 FlowNet-2 official repository can be found at https://github.com/NVIDIA/flownet2-pytorch.

4 RAFT official repository can be found at https://github.com/princeton-vl/RAFT.

5 Video of qualitative results can be found: https://www.youtube.com/watch?v=c0Xm7A_TiMQ.

## Declarations

### Code availability

Code for this paper can be found at https://github.com/labdeeman7/deepFlowFieldsForFetoscopeMosaicing.
