# Correction to: Robust fetoscopic mosaicking from deep learned flow fields

**DOI:** 10.1007/s11548-023-03018-6

**Published:** 2023-10-03

**Authors:** Oluwatosin Alabi, Sophia Bano, Francisco Vasconcelos, Anna L. David, Jan Deprest, Danail Stoyanov

**Affiliations:** 1 University of Bordeaux, Bordeaux, France; 2 Wellcome/EPSRC Centre for Interventional and Surgical Sciences (WEISS) and Department of Computer Science, University College London, London, UK; 3 Elizabeth Garrett Anderson Institute for Women’s Health, University College London, London, UK; 4 NIHR University College London Hospitals Biomedical Research Centre, London, UK; 5 Department of Development and Regeneration, University Hospital KU Leuven, Leuven, Belgium

**Correction to: International Journal of Computer Assisted Radiology and Surgery (2022) 17:1125–1134** 10.1007/s11548-022-02623-1

The original version of this article unfortunately contained a mistake. The licence information was missing and should have been:

Open Access This article is licensed under a Creative Commons Attribution 4.0 International License, which permits use, sharing, adaptation, distribution and reproduction in any medium or format, as long as you give appropriate credit to the original author(s) and the source, provide a link to the Creative Commons licence, and indicate if changes were made. The images or other third party material in this article are included in the article’s Creative Commons licence, unless indicated otherwise in a credit line to the material. If material is not included in the article’s Creative Commons licence and your intended use is not permitted by statutory regulation or exceeds the permitted use, you will need to obtain permission directly from the copyright holder. To view a copy of this licence, visit http://creativecommons.org/licenses/by/4.0/.

The original article has been corrected.

